# Supplementary material for: Fecal pollution can explain antibiotic resistance gene abundances in anthropogenically impacted environments
Source: Nat Commun. 2019 Jan 8;10:80. doi: 10.1038/s41467-018-07992-3 (PMC6325112; doi:10.1038/s41467-018-07992-3)
Supplement: Supplementary file 1 — Supplementary Information [file 41467_2018_7992_MOESM1_ESM.pdf]

Fecal pollution explains antibiotic resistance gene abundances in anthropogenically impacted environments

Karkman A., Pärnänen K., Larsson D.G. J.

Supplementary figures

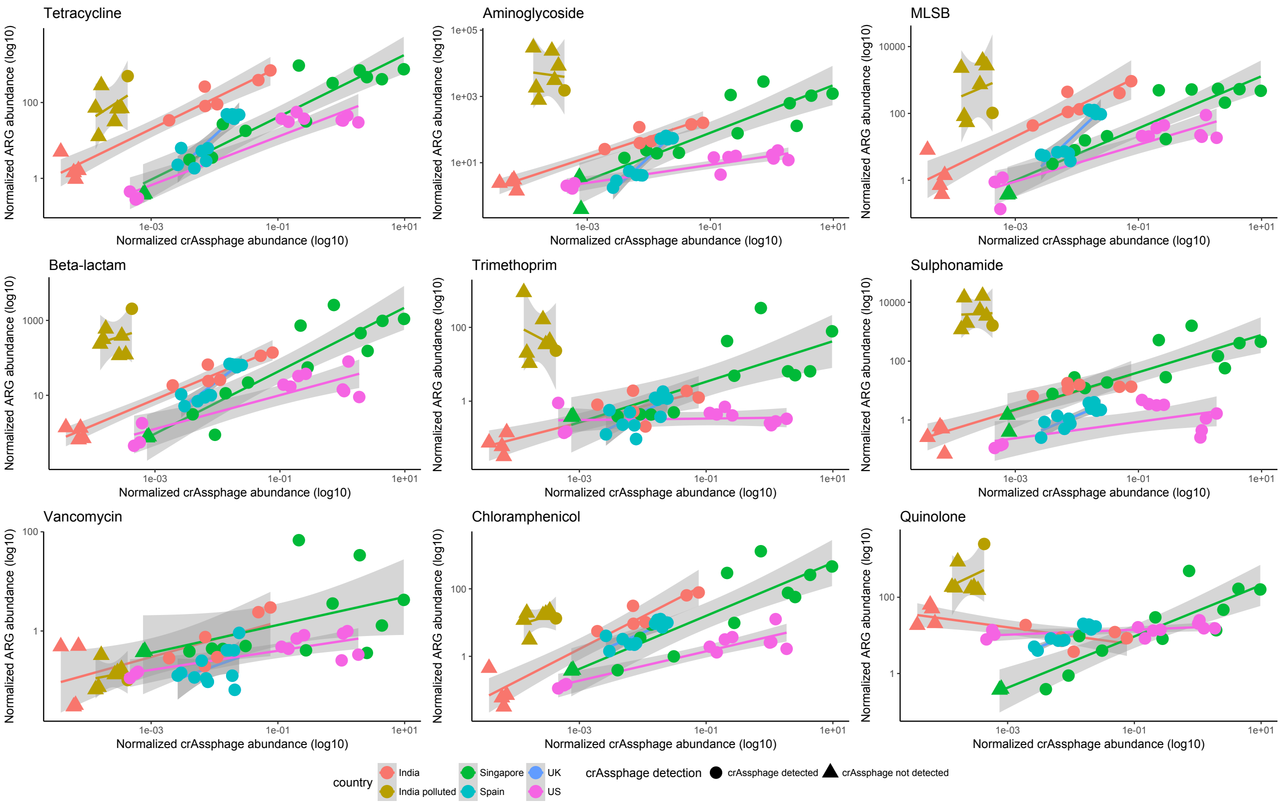

Supplementary figure 1. Correlation between abundance of different classes of ARGs and crAssphage detection in environments with pollution from WWTPs, hospitals or drug manufacturing (see main article for details). The ARG abundance and crAssphage detection were normalized with total nucleotide count in the metagenomes. For samples where we did not detect crAssphage (indicated by triangles) or ARGs, half of the detection limit (corresponding to one read mapping to crAssphage or half a count for ARGs) was used and normalized to the total nucleotide count. Smoothing curves based on linear model separately for each country are shown in colors specific for each country with 95 % confidence intervals in grey. The lack of correlation for quinolone resistance genes for two sets of metagenomes is based on one gene and the finding is discussed in the main article

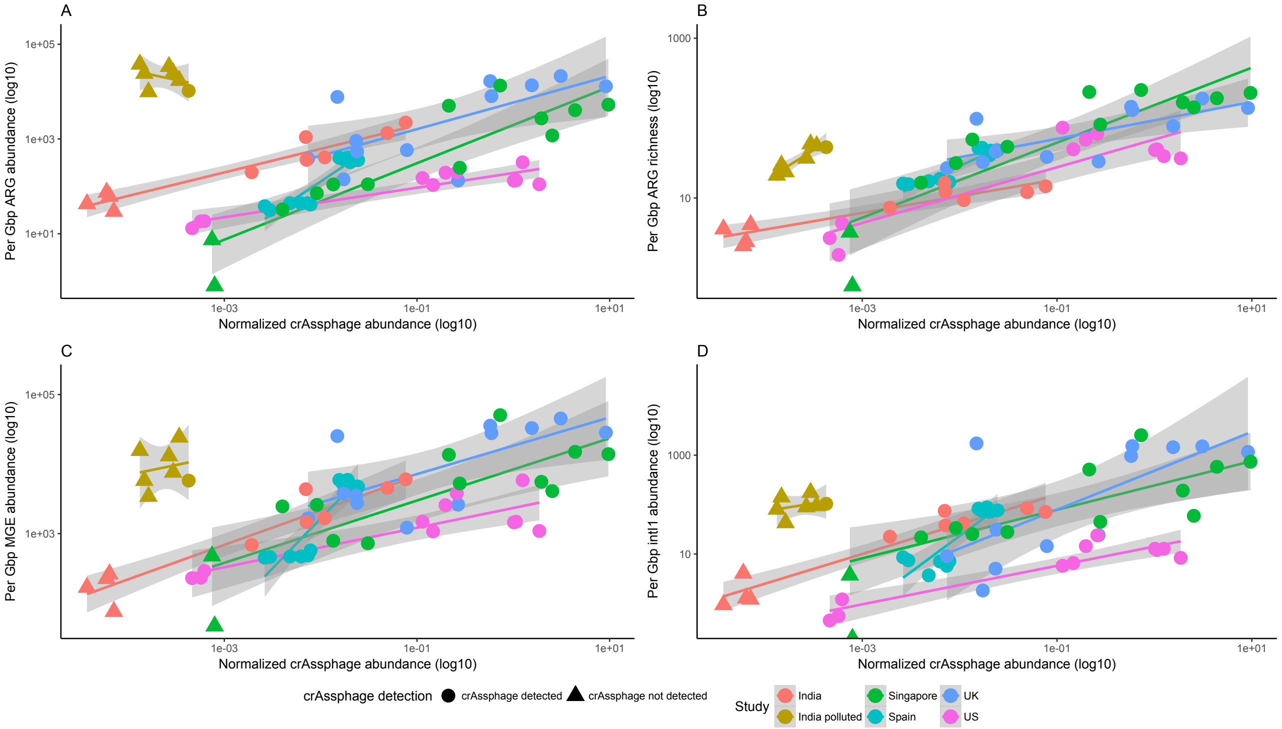

Supplementary figure 2. The correlation between crAssphage and ARG abundance (A) and richness (B) and total MGE abundance (C) and intI1 abundance (D). Smoothing curves based on linear model separately for each country are shown in colors specific for each country with 95 % confidence intervals in grey. See main article for more details.

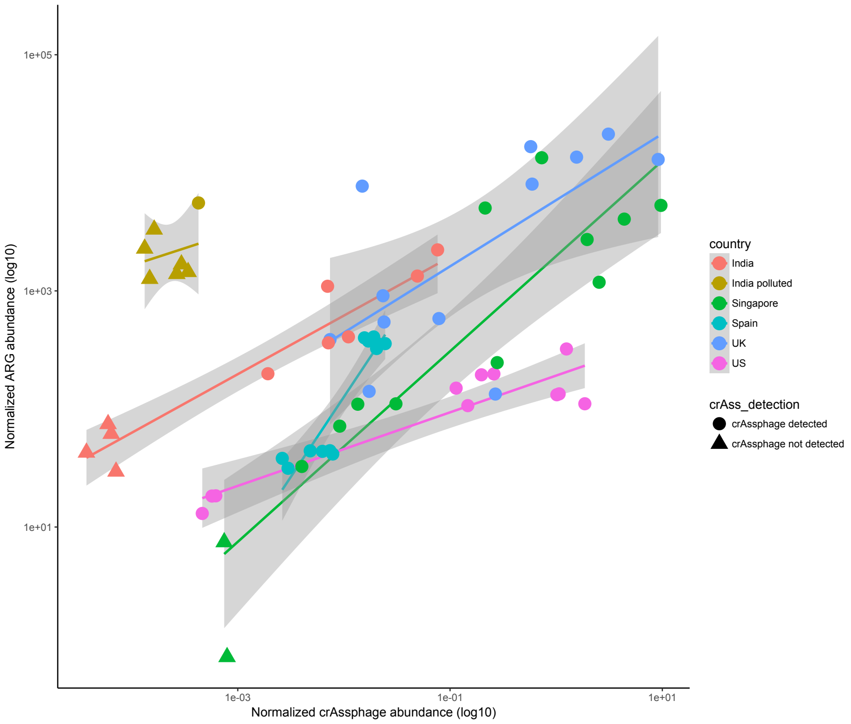

Supplementary figure 3. Correlation between ARG abundance and crAssphage detection in environments with pollution from WWTPs, hospitals or drug manufacturing (see main article). The latter is polluted with exceptionally high levels of antibiotics, and the analyses show clear selection for antibiotic resistance as the ARG abundance cannot be explained by fecal pollution. Most abundant genes possibly biased by whole genome amplification removed from the polluted Indian sediments. The ARG abundance and crAssphage detection were normalized with the total bp count in the metagenomes. Smoothing curves based on linear model separately for each country are shown in colors specific for each country with 95 % confidence intervals in grey.

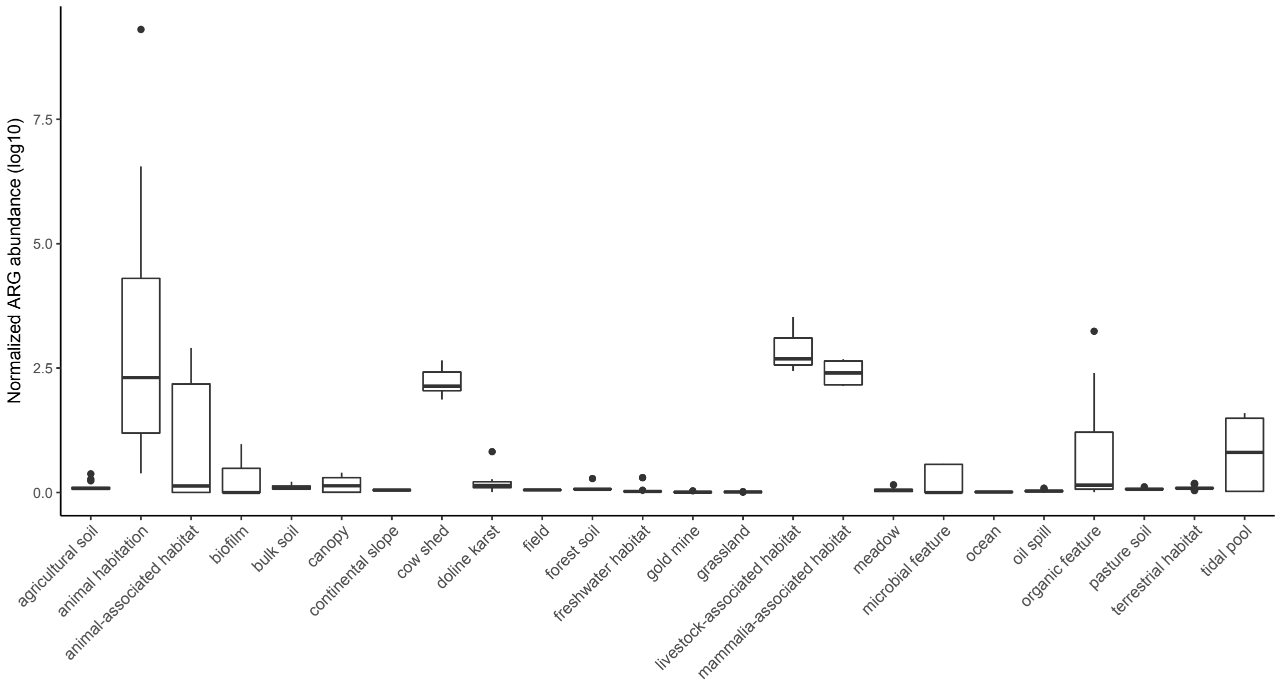

Supplementary figure 4. Antibiotic resistance gene abundance in samples from MG-RAST where crAssphage was not detected grouped by MG-RAST feature annotation.

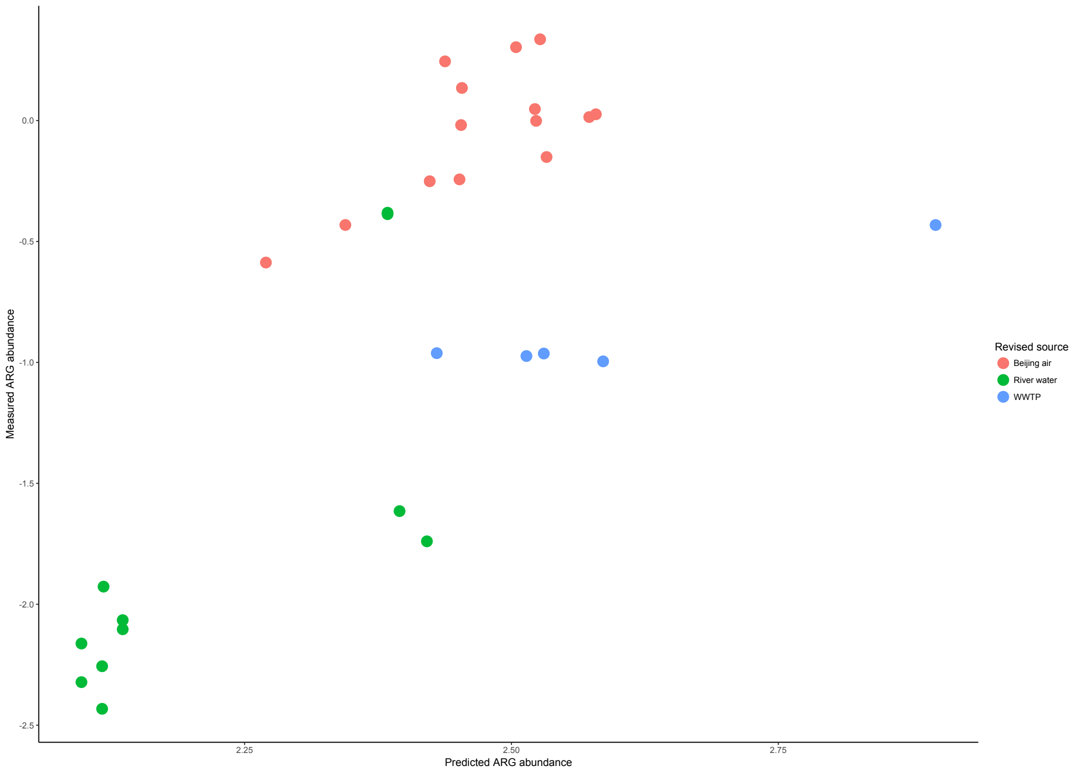

Supplementary figure 5. Antibiotic resistance gene abundance in impacted MG-RAST samples predicted from the crAssphage abundance on log10 scale using a linear model from selected studies. The correlation between measured and predicted ARG abundance is highly significant (F=34.76, Adj. R^2=0.54, $p<0.05$)

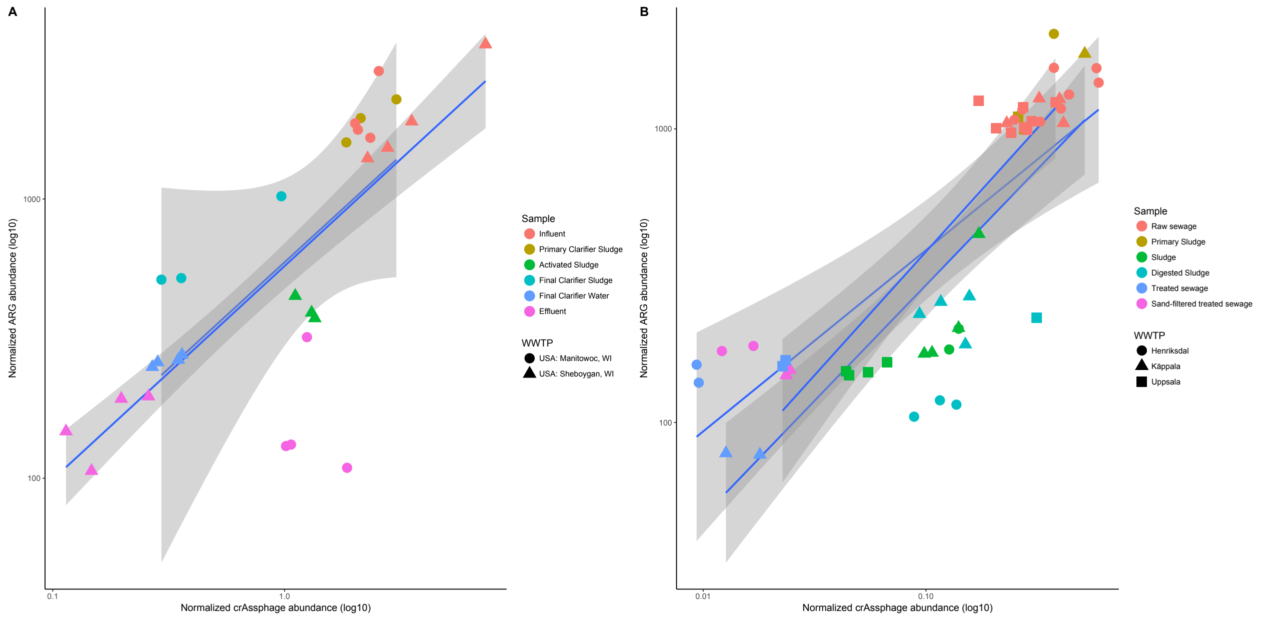

Supplementary figure 6. ARG abundance and crAssphage abundance in two US (A) and three Swedish (B) waste water treatment plants. Smoothing curves based on linear model separately for each plant are shown in blue with 95 % confidence intervals in grey.

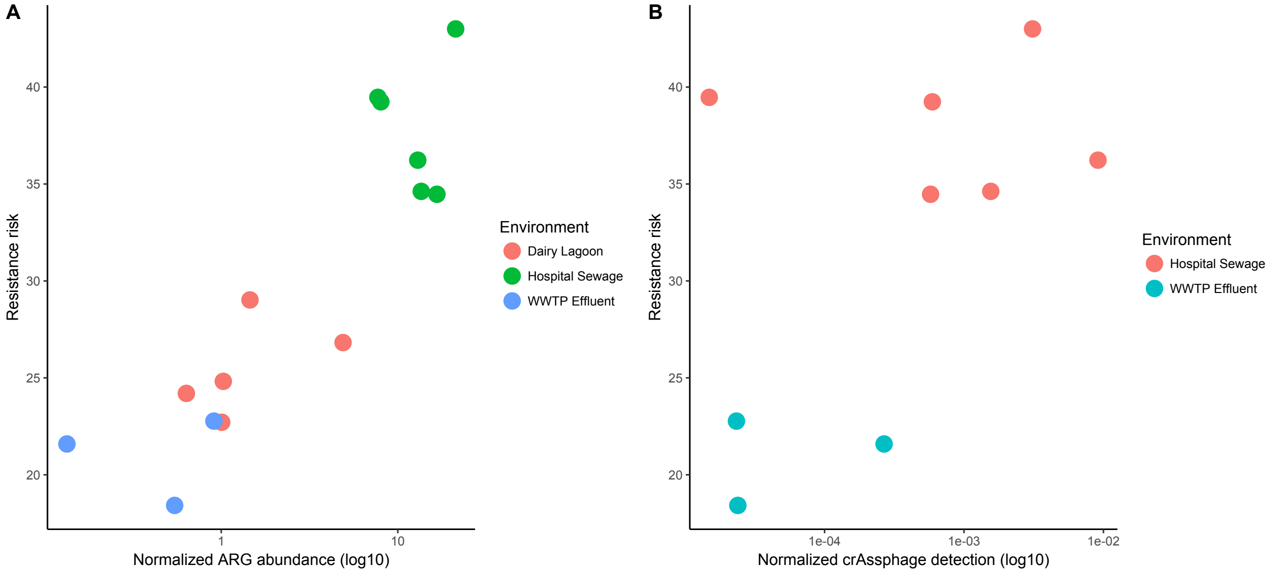

Supplementary figure 7. The correlation between resistance risk and normalized ARG abundance (A) and crAssphage detection (B). The resistance risk values were taken from Oh et al (2018) and the crAssphage detection and ARG abundance were calculated as described in the materials and methods for this study.
