# Supplementary material for: Fecal pollution can explain antibiotic resistance gene abundances in anthropogenically impacted environments
Source: Nat Commun. 2019 Jan 8;10:80. doi: 10.1038/s41467-018-07992-3 (PMC6325112; doi:10.1038/s41467-018-07992-3)
Supplement: Supplementary file 3 — Description of Additional Supplementary Files [file 41467_2018_7992_MOESM3_ESM.pdf]

Description of Additional Supplementary Files

File Name: Supplementary Data 1
Description: Data accession codes.

File Name: Supplementary Data 2
Description: Result file.

File Name: Supplementary Data 3
Description: ϕB124-14 detection.

File Name: Supplementary Data 4
Description: ARG count tables.
